# Beyond pixels: Graph filtration learning unveils new dimensions in hepatocellular carcinoma imaging

**DOI:** 10.18632/oncotarget.28635

**Published:** 2024-07-24

**Authors:** Yashbir Singh

**Keywords:** graph filtration learning, hepatocellular carcinoma, medical imaging, topological data analysis, tumor characterization

## Abstract

This editorial explores the emerging role of Graph Filtration Learning (GFL) in revolutionizing Hepatocellular carcinoma (HCC) imaging analysis. As traditional pixel-based methods reach their limits, GFL offers a novel approach to capture complex topological features in medical images. By representing imaging data as graphs and leveraging persistent homology, GFL unveils new dimensions of information that were previously inaccessible. This paradigm shift holds promise for enhancing HCC diagnosis, treatment planning, and prognostication. We discuss the principles of GFL, its potential applications in HCC imaging, and the challenges in translating this innovative technique into clinical practice.

In medical imaging, our understanding of hepatocellular carcinoma (HCC) has long been constrained by the limitations of pixel-based analysis [1]. Traditional methods, while valuable, often struggle to capture the full complexity of tumor heterogeneity, vascular patterns, and tissue architecture that characterize this aggressive liver cancer [1]. However, a new frontier is emerging in image analysis – one that promises to take us beyond pixels and into topological features and relational data structures. This frontier is Graph Filtration Learning (GFL) [2], a cutting-edge approach that can unveil new dimensions in HCC imaging and revolutionize our ability to diagnose, treat, and understand this challenging disease.

## The promise of graph filtration learning

At its core, GFL represents a paradigm shift in how we approach medical image analysis. Rather than treating images as mere collections of pixels, GFL transforms them into rich, interconnected graphs [2]. Each pixel or voxel becomes a node in this framework, with edges connecting neighboring elements. This graph representation allows us to capture not just intensity values but also spatial relationships and structural patterns within the image. The true power of GFL lies in its ability to learn meaningful filtrations on these graphs. A filtration assigns importance or relevance to different parts of the graph [2]. Through advanced machine learning techniques, GFL can automatically discover optimal filtrations highlighting clinically relevant features in HCC images. These learned filtrations serve as a foundation for applying persistent homology – a method from topological data analysis that captures multi-scale structural information about the data [3]. GFL offers a unique lens through which to view HCC imaging data by combining graph representations, learned filtrations, and persistent homology. It allows us to extract topological signatures that encode information about tumor shape, vascular networks, and tissue organization in ways that traditional pixel-based methods cannot match.

## New dimensions in HCC imaging

The application of GFL to HCC imaging unveils several exciting avenues for advancement. By capturing subtle topological features of HCC lesions, GFL enhances tumor characterization, potentially improving differentiation between benign and malignant liver nodules and offering new prognostic indicators based on tumor architecture patterns [2, 4]. GFL’s ability to represent and analyze complex network structures makes it particularly well-suited for vascular network analysis. It is crucial for understanding the blood supply of these highly vascularized tumors and predicting treatment response to interventions like transarterial chemoembolization. The multi-scale approach of GFL could provide new insights into intratumoral heterogeneity, a significant challenge in HCC management, by mapping the spatial distribution of different cell populations within a tumor [4]. This could guide more precise biopsy targeting and inform personalized treatment strategies. GFL’s capacity to capture persistent topological features across different imaging [5] time points offers robust methods for longitudinal tracking of tumor evolution and treatment response, particularly valuable in assessing systemic therapy efficacy and monitoring for recurrence after local treatments. Furthermore, GFL-derived features could complement traditional radiomic features, enriching the data for building predictive models. This synergy between topological and conventional image analysis has the potential to lead to more accurate and interpretable AI models for HCC management, ultimately improving patient care and outcomes.

## Challenges and future directions

While GFL holds immense potential HCC imaging, several challenges must be addressed to realize its promise fully. The computational complexity of GFL, particularly in persistent homology computations, necessitates algorithm optimization for large-scale medical imaging datasets to ensure clinical feasibility. Ensuring the interpretability of GFL-derived features is crucial for gaining clinical trust and understanding their biological significance. Developing standardized protocols for applying GFL across different imaging modalities and scanner types is essential for reproducible results. Rigorous clinical studies are needed to demonstrate the added value of GFL-derived features in improving patient outcomes across various aspects of HCC management. Integrating GFL analysis into existing radiology workflows and decision support systems is vital for its practical adoption. Overcoming these challenges will be critical in translating the innovative GFL technique into clinical practice, ultimately enhancing HCC diagnosis, treatment planning, and prognostication.

Looking ahead, several exciting directions for future research emerge:

## Multi-modal integration

Exploring how GFL can be applied to multi-modal imaging data (e.g., combining CT, MRI, and PET) could provide even richer characterizations of HCC lesions.

## Radiogenomics

Investigating correlations between GFL-derived topological features and genomic profiles of HCC tumors could uncover new biomarkers and advance our understanding of tumor biology.

## Treatment planning

Developing GFL-based models to predict treatment response and optimize interventional strategies, such as guiding ablation margins or planning radiation therapy.

## Early detection

Exploring the potential of GFL in detecting subtle changes in liver parenchyma that may indicate early-stage HCC or predict its development in high-risk patients.

## Explainable AI

Advancing methods to visualize and interpret the topological features captured by GFL, making them more accessible and actionable for clinicians.

As we stand on the brink of a new era in medical imaging analysis, GFL emerges as a powerful tool to take us beyond the limitations of pixel-based approaches. By unveiling new dimensions in HCC imaging, GFL promises to enhance our understanding of tumor biology, improve diagnostic accuracy, and guide more personalized treatment strategies. While challenges remain in translating this innovative technique into clinical practice, the potential benefits for HCC patients are immense. The journey beyond pixels is just beginning, and GFL is lighting the way. As researchers, clinicians, and data scientists collaborate to refine and validate this approach, we can look forward to a future where the hidden topological features of HCC are no longer beyond our grasp. In this future, every image tells a richer story, and every patient benefits from a deeper understanding of their unique disease. The dimensions unveiled by GFL may be the key to unlocking new horizons in HCC management, offering hope for improved outcomes in the face of this formidable cancer.
